# Author Correction: All-optical implementation of collision-based evolutions of open quantum systems

**DOI:** 10.1038/s41598-020-61337-z

**Published:** 2020-03-04

**Authors:** Álvaro Cuevas, Andrea Geraldi, Carlo Liorni, Luís Diego Bonavena, Antonella De Pasquale, Fabio Sciarrino, Vittorio Giovannetti, Paolo Mataloni

**Affiliations:** 1 Department of Physics, University of Rome La Sapienza, Piazzale Aldo Moro 5, 00185 Rome, Italy; 2 Heinrich-Heine Universität, Institut für Theoretische Physik III, Universitätstraße 1, 40225 Düsseldorf, Germany; 3 Department of Physics, University of Florence, Via G. Sansone 1, I-50019 Sesto Fiorentino, Florence Italy; 4 INFN Sezione di Firenze, Via G. Sansone 1, I-50019 Sesto Fiorentino, Florence Italy; 5 NEST, Scuola Normale Superiore and Istituto Nanoscienze, Piazza dei Cavalieri 7, 56126 Pisa, Italy

Correction to: *Scientific Reports* 10.1038/s41598-019-39832-9, published online 01 March 2019

This Article contains an error in the legend of Figure 5, where ‘*θ* = *π*/4’ should read ‘*θ* = *π*/2’.

